# Echocardiographic myocardial work in pre-adolescent male basketball players: a comparison with cardiopulmonary exercise test-derived aerobic capacity

**DOI:** 10.3389/fphys.2022.913623

**Published:** 2022-07-22

**Authors:** Kewei Zhao, Yu Liu, Lili Dong, Binghong Gao

**Affiliations:** ^1^ High Performance Research Center, China Institute of Sport Science, Beijing, China; ^2^ Department of Echocardiography, Zhongshan Hospital, Fudan University, Shanghai, China; ^3^ Department of Cardiology, Zhongshan Hospital, Fudan University, Shanghai, China; ^4^ Shanghai Institute of Cardiovascular Diseases, Shanghai, China; ^5^ School of Physical Education and Sport Training, Shanghai University of Sport, Shanghai, China

**Keywords:** myocardial work, young athletes, athlete’s heart, exercise test, speckle tracking echocardiography

## Abstract

**Background:** Pressure-strain loop (PSL) analysis provides a novel, less load-dependent non-invasive method to quantify myocardial work and demonstrates a significant correlation with the contractile reserve in adult athletes. We aim to validate PSL-derived markers in characterizing LV function in pre-adolescent basketball players by comparing results before and after the cardiopulmonary exercise test (CPX) and explore its association with CPX-derived aerobic capacity.

**Methods:** Cardiac morphology and function in 20 pre-adolescent basketball players were assessed at 9.7 years old (9.7 ± 1.1 year) before and after cardiopulmonary exercise testing. Echocardiography was performed in all subjects, including two-dimensional speckle-tracking echocardiography (STE). Simultaneous brachial-cuff-measured blood pressure was recorded to perform PSL analysis.

**Results:** Nineteen subjects were included in the final analysis. Exercise training in pre-adolescent males was associated with lower global work index (GWI) and global work efficiency (GWE) at rest. GWE at stress was significantly correlated with VO_2_max and peak O_2_ pulse (*p* = 0.0122, *r* = 0.56; *p* = 0.00122, *r* = 0.69, respectively). When indexed by body mass, GWI and GWE both significantly correlated with relative VO_2_max (*p* = 0.0086 and 0.0011 respectively, *r* = 0.58 and 0.69 respectively); GWI and GWE at baseline and stress were all significantly correlated with peak O_2_ pulse (GWI at baseline, *p* **<** 0.0001, *r* = −0.90; GWE at baseline, *p* **<** 0.0001, *r* = −0.89; GWI at stress, *p* **=** 0.0289, *r* = −0.50; GWE at stress, *p* **<** 0.0001, *r* = −0.83).

**Conclusion:** PSL-analysis-derived GWI and GWE at rest indexed by body mass are associated with cardiopulmonary exercise test-derived peak oxygen consumption and oxygen pulse in pre-adolescent athletes.

## Introduction

Cardiac morphological and functional remodeling in response to long-term training includes increased left ventricular (LV) wall thickness, LV mass, increased LV and RV right ventricular (RV) chamber size (Huston et al., 1985) and low-normal range of systolic function as measured by ejection fraction (Baggish and Wood, 2011; Prior and La Gerche, 2012; Lakatos et al., 2020). Similar morphological changes have also been reported in pre-adolescent children (Ayabakan et al., 2006; Bjerring et al., 2018) but previous studies have given inconsistent findings on the change of systolic function in trained young athletes (Obert et al., 2009; McClean et al., 2018; Unnithan et al., 2018; Bjerring et al., 2019; Li et al., 2020), even with similar deformational parameters. This may be attributed to the load dependency of conventional echocardiographic parameters to quantify LV function.

Pressure-strain loop (PSL) analysis provides a novel non-invasive method to quantify myocardial work, which takes into consideration the impact of afterload when evaluating LV mechanics (Russell et al., 2012) and thus may provide better estimates of LV systolic function. It has already been tested under several pathological conditions (Chan et al., 2019; El Mahdiui et al., 2019; Hiemstra et al., 2020; Sengupta et al., 2020; Jaglan et al., 2021) and proved feasible in a healthy paediatric population (Pham et al., 2021). Besides, PSL markers also demonstrated a significant correlation with peak oxygen consumption (VO_2_max) in adult athletes and were suggested to be good predictors of the contractile reserve under stress (Tokodi et al., 2021; D'Andrea et al., 2020). The aim of this cross-sectional study was to evaluate LV function with PSL analysis in a group of pre-adolescent basketball players during a cardiopulmonary exercise test (CPX) and to explore the relation of these PSL-derived indices to CPX-derived aerobic capacity (both VO_2_max and VO_2_max/kg).

## Methods

In August 2019, 20 basketball players between 8 and 10 years old were recruited during their routine cardiopulmonary exercise test (CPX). They have been practicing basketball 5 times a week for 2 h each time for 18 months. They trained on a children’s basketball court, which was 22 m long and 12 m wide, with 2.6 m high baskets and a size five ball (22 cm diameter) for training. They train mainly with games and individual technical training with medium intensity. From Monday to Thursday afternoons are training and Friday afternoons are games, where there is no position differentiation between players on the court. All subjects gave written informed consent in accordance with the Declaration of Helsinki. The protocol was approved by the “Science Research Ethics Committee at the Shanghai University of Sport.” All athletes’ parents were informed about the protocol of this study, which was outlined in an information letter. No data collection took place without parents’ consent.

### Transthoracic echocardiography

Participants underwent a complete two-dimensional echocardiographic study (2DE) at baseline and a focused scan immediately after CPX on Vivid iq (GE, Vingmed, Horten, Norway) with a 2.5 MHz phased array transducer (M5Sc). Cuff-measured blood pressure was recorded concomitantly with the scanning session. Data were digitally stored for offline analysis (EchoPAC, GE, Vingmed). From 2DE, LV dimensions, ejection fraction by Simpson’s biplane method, and LV diastolic function parameters were assessed. Left atrial (LA) volume was measured using the biplane method. Right atrial (RA) area and RV function parameters were assessed in the RV-focused apical four-chamber view. Parameters were measured according to the latest recommendations (Lang et al., 2015; Mitchell et al., 2019). LV mass was calculated using the Devereux formula. Relative wall thickness (RWT) was calculated as 2 times the posterior LV wall thickness divided by LV end-diastolic diameter.

### Myocardial work

LV myocardial work was calculated by integrating longitudinal strain and brachial-cuff-measured blood pressure, as previously described by Russel et al. (Russell et al., 2012). LV longitudinal strain was measured using speckle-tracking analysis in the standard two-, three-, and four-chamber apical views. The region of interest was automatically created and manually adjusted when necessary. LV global longitudinal strain (GLS) was then calculated by averaging the peak longitudinal strain in 17 segments from the three apical views. The peak systolic LV pressure was assumed to equal the peak arterial systolic pressure. A non-invasive LV pressure-strain curve was then constructed using EchoPAC (ver.203) and adjusted according to the duration of the ejection and isovolumetric phases, which were defined by the opening and closure of the mitral and aortic valves. The following parameters were calculated as with previous studies (Chan et al., 2019; El Mahdiui et al., 2019; Manganaro et al., 2019; Yedidya et al., 2021):

Global myocardial work index (GWI): the total work within the area of the LV PSL between mitral valve closure and mitral valve opening.

Constructive myocardial work (CW): shortening of the myocytes during systole adding lengthening of the myocytes during isovolumic relaxation.

Wasted myocardial work (WW): lengthening of myocytes during systole (absorbing work done by other segments) adding shortening during the isovolumic relaxation phase (not contributing to ejection).

**FIGURE 1:**
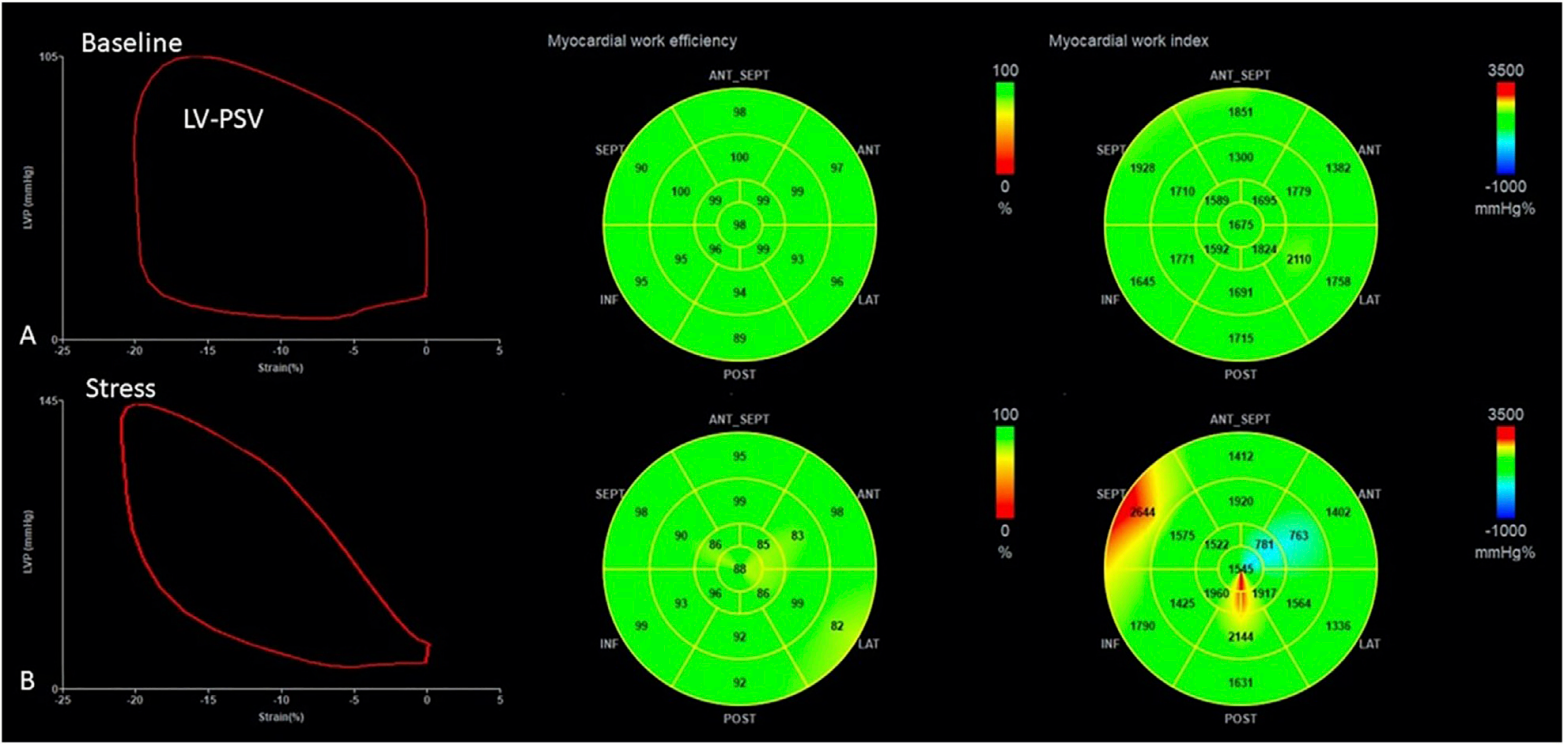
Measurement of myocardial work parameters by 2D echocardiography both at baseline **(A)** and during stress **(B)**. The left panel demonstrates the generated LV pressure–strain loop; The middle and the right panels are bull’s eye plot of GWE and GWI, respectively. GWE, global work efficiency; GWI, global work index.

Global cardiac work efficiency (GWE): Cardiac work efficiency was expressed as CW/(CW + WW) × 100% per segment and GWE as an average of all segmental values (Figure 1).

### Cardiopulmonary exercise testing

Maximal oxygen uptake (VO_2_max) was determined by a ramp incremental exercise test (Michalik et al., 2019), with a starting load of 30 W, a continuous incremental load of 1 W every 3 s (20 W/min), maintaining a speed of 60 rpm to exhaustion on a bicycle ergometer (Lode Excalibur, Netherlands).

When the participant could no longer complete the desired workload, the test was terminated. Oxygen uptake was measured continuously with an automated system (Cortex Matamax 3B, Germany). The exercise test was accepted as maximal if the majority of the following termination criteria were met: respiratory exchange ratio greater than 1.0, heart rate greater than 200 beats/minute, display of indicators of a maximal effort such as sweating and, despite strong verbal encouragement, the participant was unable or unwilling to continue.

### Statistical analysis

The Shapiro-Wilk test for normality was performed on all data sets. Continuous data were expressed as mean ± SD or as median (interquartile range) according to data distribution. CPX data were compared with their predicted values through either paired *t*-test or Wilcoxon signed rank test depending on data distribution. Z-test for single sample was performed to compare echocardiographic indices and relative VO_2_max of this cohort with reference data of normal children whose population mean and variance was known (Rosner, 2011). Specifically, the reference values of conventional echocardiographic measurements were taken from a study of the local children’s hospital for a similar age group not stratified by gender, as gender differences in this age group (age under 11) for these measurements were not statistically significant (Deng et al., 2006). The control value of LV GLS was adopted from the study of Koopman LP et al. for a similar age group not stratified by gender, as measurements of LV GLS were similar for both genders (Koopman et al., 2019). The control values of myocardial work indices were taken as the mean and variance of the general pediatric population not stratified by age or gender, as these factors may not affect GWI or GWE (Pham et al., 2021). Comparison between parameters at baseline and during stress for continuous variables was made by paired *t*-test for normally distributed data. Correlations between VO_2_max and both conventional LV systolic function parameters and PSL-derived myocardial work markers were evaluated by Pearson’s correlation coefficient. The Z-tests were performed manually and all other statistical analyses were performed using SPSS version 20.0 (SPSS, Chicago, IL), and a value of *p* < 0.05 was considered statistically significant.

## Results

### Demographics and cardiopulmonary exercise testing data

All participants completed echocardiographic scans and CPX but one was excluded from the final analysis due to poor image quality. The basic demographic characteristics of the athletes are summarized in Table 1 and the results of CPX are presented in Table 2. Nearly all CPX variables except for maximal workload were significantly lower than their predicted values by Neder formula, which was derived using subjects over 20 years old. This was within expectation as the participants’ heart were still undergoing maturation.

**TABLE 1:** Demographic characteristics of the athletes.

Variable	Value
Age (year)	9.9(2.2)
Height (cm)	147.5 ± 9.4
Body mass (kg)	38.2 ± 7.9
BSA (m^2^)	1.24 ± 0.16
Duration of participation (year)	2.4(0.5)
SV (ml)	48.5 ± 8.9
HR (bpm)	80.7 ± 13.0
CO (L/min)	3.906 ± 0.962

BSA, body surface area; SV, stroke volume; HR, heart rate; CO, cardiac output.

**TABLE 2:** Cardiopulmonary exercise testing variables and their predicted values of the athletes.

Variable	Value	Predicted Value (Neder formula (Neder et al., 1999))	*p* value
VO_2_peak (L/min)	1.967 ± 0.328	2.397 ± 0.172	**< 0.0001**
Percentage of predicted VO_2_peak (%)	81.6 ± 8.4	—	—
Relative VO_2_peak (ml/min/kg)	51.67 ± 4.32	—	—
Maximum workload (Watt)	176.4 ± 23.7	163.5 ± 16.5	0.0955
Percentage of predicted maximum workload (%)	109.3 ± 20.3	—	—
HRpeak (bpm)	195 ± 10	202(2)	**0.0046**
Percentage of predicted HRpeak (%)	96.2 ± 4.7	—	—
Oxygen pulse (mLO_2_/beat)	9.9 ± 1.5	12.7 ± 0.7	**< 0.0001**
Percentage of predicted oxygen pulse (%)	77.7 ± 8.5	—	—
RERpeak	1.03 ± 05	—	—
VEpeak (L/min)	75.8 ± 13.4	136.4(2.2)	**< 0.0001**
VE/VO_2_	34.7 (4.5)	—	—
VE/VCO_2_ slope	33.1 (2.8)	—	—

VO_2_peak, peak oxygen uptake; HRpeak, peak heart rate; RERpeak, peak respiratory exchange ratio; VEpeak, peak minute ventilation; VE/VO_2_, ventilatory equivalents of oxygen; VE/VCO_2_ slope, slope of minute ventilation versus carbon dioxide below the onset of terminal hyperventilation.

Bold values indicate a p < 0.05. Predicted Values are calculated using gender-specific Neder formula.

The relative VO_2_max of the study population was significantly higher than their age-matched peers (reference population mean ± SD = 36.4 ± 5.5 ml/min/kg, Z score = 12.10189, *p* < 0.00001) (Yu et al., 2019), which was in agreement with their regular training.

### Conventional echocardiography findings

All participants reported more than 9 h of weekly exercise and were classified as active athletes without further subdivision. Data from 2DE and the Z-test results are summarized in Table 3. The study cohort demonstrated enlarged left ventricle dimensions and slightly-decreased left ventricular ejection fraction with preserved LV-GLS, which were concordant with previous studies on cardiac remodelling under long training.

**TABLE 3:** 2D echocardiographic parameters.

Variable	Value	References Value	Z-score	*p* value
IVSd^a^ (mm)	6.6 ± 0.6	5.8 ± 0.7	4.9816	**< 0.00001**
LVIDd^a^ (mm)	42.3 ± 2.8	39.4 ± 2.7	4.68178	**< 0.00001**
LVPWd^a^ (mm)	6.4 ± 0.9	5.3 ± 0.9	6.8497	**< 0.00001**
LVIDs^a^ (mm)	26.7 ± 2.2	24.9 ± 2.6	3.0177	**0.00252**
LV mass (g)	83.6 ± 16.5	—	—	—
LVMi (g/m^2^)	67.7 ± 10.4	—	—	—
RWT	0.30 ± 0.05	—	—	—
EDV^a^ (ml)	73.4 ± 19.8	68.1 ± 11.0	2.1002	**0.03572**
EDVi (ml/m^2^)	59.4 ± 13.8	—	—	—
ESV (ml)	29.0(11.0)	—	—	—
ESVi (ml/m^2^)	24.1(6.9)	—	—	—
EF (%)	59(5)	67 ± 6	-14.2361	**< 0.00001**
LV-GLS^b^ (%)	−21.2 ± 1.3	−21.4 ± 2.3	0.37903	0.70394
CI (L/min/m^2^)	3.144 ± 0.559	—	—	—
LAVd (ml)	32.3 ± 12.0	—	—	—
LAVs (ml)	11.4 ± 4.4	—	—	—
E (cm/s)	104.6 ± 22.4	—	—	—
A (cm/s)	55.4 ± 13.4	—	—	—
E/A	1.96 ± 0.51	—	—	—
DT (ms)	150(26)	—	—	—
mitral S' (cm/s)	10.6 ± 1.8	—	—	—
e' (cm/s)	16.7 ± 1.9	—	—	—
E/e'	6.3 ± 1.4	—	—	—
EDA (cm^2^)	16.0 ± 2.8	—	—	—
ESA (cm^2^)	7.6 ± 2.2	—	—	—
RAAd (cm^2^)	10.6 ± 2.5	—	—	—
RAAs (cm^2^)	7.0 ± 1.7	—	—	—
TAPSE (mm)	19.0(4.0)	—	—	—
tricuspid S' (cm/s)	11.2 ± 1.3	—	—	—
RVFWSL (%)	−27.6 ± 3.0	—	—	—
RVGLS (%)	−23.0 ± 2.4	—	—	—
GWI^c^ (mmHg%)	1,524 ± 133.8	1,688 ± 219	−3.2642	**0.00112**
GWE^c^ (%)	92.2 ± 3.4	96.5 ± 1.4	−13.38805	**< 0.00001**

a Reference values of these variables are from the study by Deng et al. (2006).

b Reference value of this variable is from the study by Koopman et al. (2019).

c Reference values of these variables are from the study by Pham et al. (2021).

IVSd, interventricular septum at end diastole; LVIDd, left ventricular internal diameter at end diastole; LVPWd, left ventricular posterior wall at end diastole; LVIDs, left ventricular internal diameter at end systole; LV, left ventricle; LVMi, left ventricular mass index; RWT, relative wall thickness; EDV, end-diastolic volume; EDVi, end-diastolic volume index; ESV, end-systolic volume; ESVi, end-systolic volume index; EF, ejection fraction; LV-GLS, left ventricular global longitudinal strain; CI, cardiac index; LAVd, left atrial volume at end systole; LAVs, left atrial volume at end diastole; E, velocity of mitral inflow E wave; A, velocity of mitral inflow A wave; DT, deceleration time; s’, systolic velocity of mitral/tricuspid annulus; e’, velocity of mitral annular diastolic e’ wave; EDA, end-diastolic area; ESA, end-systolic area; RAAd, right atrial area at end systole; RAAs, right atrial area at end diastole; TAPSE, tricuspid annular plane systolic excursion; RVFWSL, right ventricular free wall longitudinal strain; RVGLS, right ventricular global longitudinal strain; GWI, global work index; GWE, global work efficiency.

Bold values indicate a p < 0.05.

### Myocardial work indices

**FIGURE 2:**
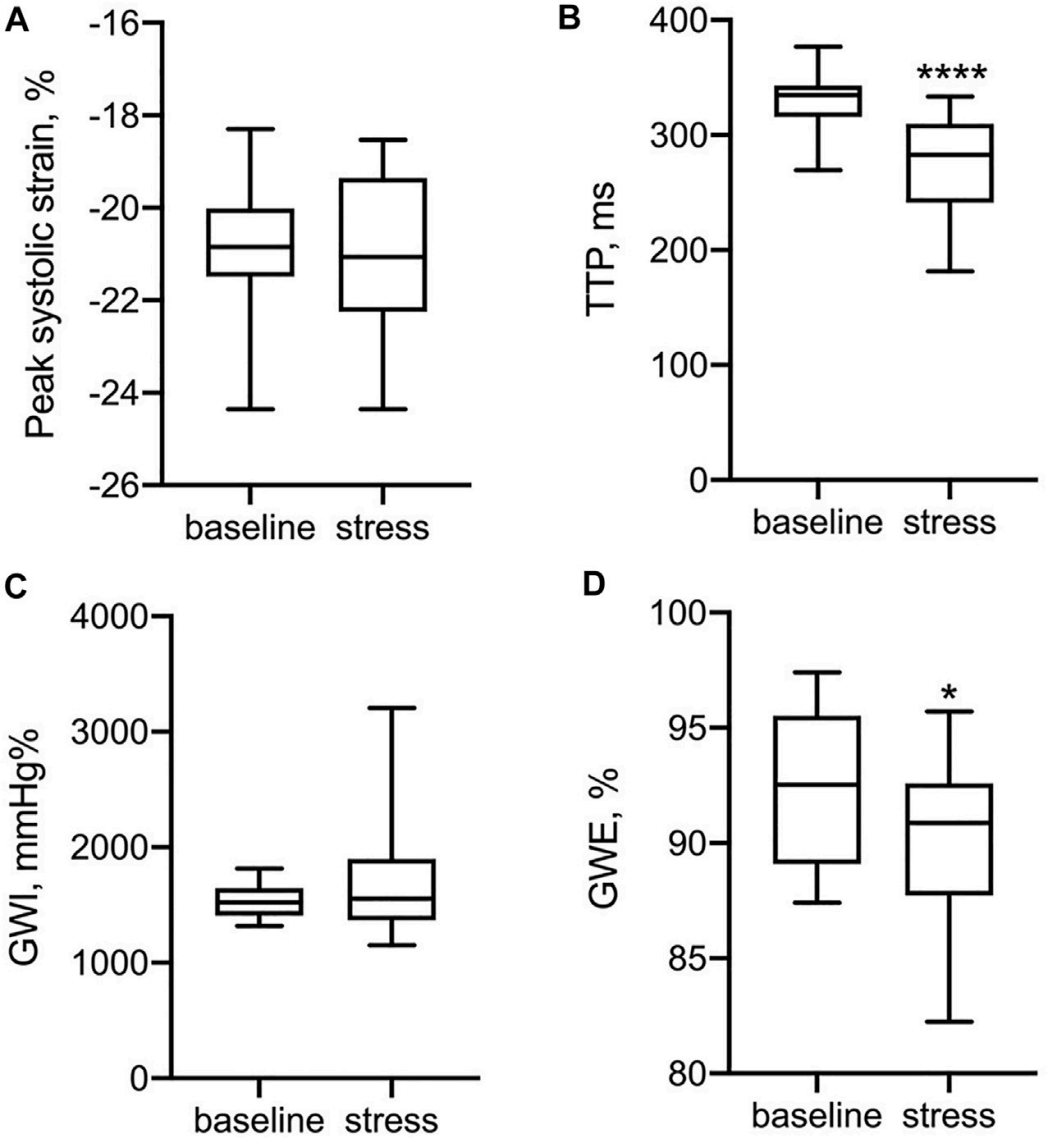
Comparison of LV functional parameters between baseline and stress status.

Exercise training in pre-adolescent males was associated with lower GWI and GWE at rest (Table 3). In Figure 2, LV functional parameters were compared between baseline and stress status. As expected, the time to peak strain (TTP) was significantly shorter during the stress test (*p* < 0.0001) due to increased heart rate, while peak systolic strain was not significantly different after CPX, which could be explained by the heterogeneous stress status each participant reached. For myocardial work indices, GWE rather than GWI changed significantly (*p* = 0.035).

### Correlation of LV functional parameters with peak oxygen uptake

Among conventional LV functional parameters, only stroke volume (SV) and cardiac output (CO) were significantly correlated with VO_2_max (*p* = 0.0107 and 0.005 respectively, *r* = 0.57 and 0.62 respectively) but ejection fraction, global longitudinal strain and fractional shortening were not (all *p* > 0.05). GWE at stress was significantly correlated with VO_2_max (*p* = 0.0122, *r* = 0.56). When indexed by body mass, GWI and GWE both significantly correlated with relative VO_2_max (*p* = 0.0086 and 0.0011 respectively, *r* = 0.58 and 0.69 respectively).

### Correlation of LV functional parameters with peak oxygen pulse

SV and CO were significantly correlated with peak oxygen pulse (O_2_ pulse) (*p* = 0.0356 and 0.0096 respectively, *r* = 0.48 and 0.58 respectively) but ejection fraction, global longitudinal strain and fractional shortening were not (all *p* > 0.05). GWE at stress was significantly correlated with peak O_2_ pulse (*p* = 0.00122, r = 0.69). When indexed by body mass, GWI and GWE at baseline and stress were all significantly correlated with peak O_2_ pulse (GWI at baseline, *p* **<** 0.0001, *r* = −0.90; GWE at baseline, *p* **<** 0.0001, *r* = −0.89; GWI at stress, *p* **=** 0.0289, *r* = −0.50; GWE at stress, *p* **<** 0.0001, *r* = −0.83).

## Discussion

The main findings of the present study can be summarized as follows: 1) only GWE at stress but no conventional LV systolic parameters or MW indices at rest correlates with VO_2_max; 2) GWI and GWE indexed by body mass correlates with relative VO_2_max in addition to SV, CO, age and BSA; 3) GWI and GWE indexed by body mass correlates with oxygen pulse in addition to SV and CO.

The morphological cardiac remodelling in pre-adolescent athletes has been well documented and confirmed by pooled analysis (McClean et al., 2018). However, the adaption of the LV systolic function in trained young athletes remains controversial. Previous studies engaging conventional echocardiographic parameters of LV systolic function found no difference between athletes and controls in pre-adolescents (Ayabakan et al., 2006; Bjerring et al., 2018), and deformation parameters such as global longitudinal strain (GLS) also gave similar negative findings (Bjerring et al., 2019). However, there are also studies that demonstrated slightly decreased LV GLS in young athletes (Li et al., 2020) which is a common finding in adult athletes (Baggish and Wood, 2011).

As the “pre-adolescent athletic heart” is guaranteed to transform into the “adult athletic heart” at certain time point if there is no cessation of training, the heterogeneity of study population could be one reason for the inconsistency. The limits of conventional echocardiographic parameters of LV systolic function or GLS could be another reason as they may not be sensitive enough to detect the functional change in pre-adolescent athletes. GLS remains load dependent (Voigt and Cvijic, 2019), which might represent a limitation in case of changes in the hemodynamic conditions. Myocardial work has been introduced as a new parameter of LV function that takes into account LV deformation as well as LV afterload by constructing an LV PSL on the basis of non-invasive LV pressure (sphygmomanometric blood pressure) measurements (Russell et al., 2012).

A major finding of the current study is that GWI and GWE indexed by body mass correlate with relative VO_2_max in pre-adolescent athletes. Previous studies in adult athletes have reported the association between either GWI or GWE at rest and relative VO_2_max (Tokodi et al., 2021; D'Andrea et al., 2020). The current study is different in that the GWI and GWE alone did not demonstrate correlation with either VO_2_max or relative VO_2_max but the indexed ones did. The indexed GWI is easy to understand as GWI is a measure of all the work generated by the heart and thus is closely related to the metabolic need of the body while indexing could address the difference of body mass among individuals which may be especially important in children. However, the indexed GWE is hard to understand as it is expressed as a ratio and thus should not be subject to the difference in body mass. The current study provides evidence for predicting contractile reserve at rest with MV indices in pre-adolescent athletes but future studies with larger sample size should be performed to validate the results.

Another important finding is the correlation of MW indices to the peak oxygen pulse. Oxygen pulse expresses the volume of oxygen ejected from the ventricles with each cardiac contraction and peak O_2_ pulse has been suggested to represent maximal working capacity (Wasserman et al., 1967). Current guideline recommends peak O_2_ pulse as an important measure obtained from CPX (Paridon et al., 2006). In the current study, GWI and GWE indexed by body mass correlate with peak O_2_ pulse stronger than with relative VO_2_max. As the calculation of GWI and GWE is based on single heartbeat, the better correlation with peak O_2_ pulse could be achieved after excluding the effect of heart rate. This finding also adds to the evidence for predicting aerobic capacity with MV indices in pre-adolescent athletes.

Another interesting finding in the current study is that GWI of pre-adolescent males was lower than normal children. GWI has been demonstrated to be similar or higher in athletes than in sedentary controls in adults. Jain et al. (Sengupta et al., 2020) performed MW analysis in 24 half-marathon runners: all athletes had increase in LV volume and a mild reduction in peak GLS, associated with a normal value of GWI. While in a study of 20 elite swimmers, Tokodi et al. showed that regular exercise training was associated with decreased GLS but increased values of GWI at rest (Tokodi et al., 2021). The inconsistency between the current study and previous research could be explained by the sensitivity of GWI to afterload and thus its wide range of normal values that overlaps with pathological ones (Manganaro et al., 2019). In addition, lower GWI could be a characteristic change in pre-adolescent athletes. Li et al. also demonstrated in 36 professional young strength male athletes that GWI was lower in athletes than in normal controls. Future studies engaging wide spectrum of sports categories are guaranteed.

## Limitation

A major limitation of the current study is its limited sample size despite active recruitment performed by the researchers. Sensitivity tests were made by randomly excluding one subject and the major finding of the current study remained unchanged. However, it would be better validated with larger sample size.

The current study is also subject to its narrow spectrum of sport disciplines. Thus, the generalizability of the current results needs to be clarified in a larger study population. The time interval between peak stress and echocardiographic scan may cause error in peak systolic strain measurement and thus affect PSL analysis. However, given the simultaneous measurement of blood pressure and the intrinsic heterogeneity of individual stress load, such error would not significantly change our current findings.

## Conclusion

PSL-analysis-derived GWI and GWE at rest indexed by body mass are associated with cardiopulmonary exercise test-derived peak oxygen consumption and oxygen pulse in pre-adolescent athletes. Our findings support the use of PSL analysis in the evaluation of pre-adolescent athletes’ heart.

## Data Availability

The original contributions presented in the study are included in the article/Supplementary Materials, further inquiries can be directed to the corresponding authors.
